# Use of mucin like cancer associated antigen (MCA) in the management of breast cancer.

**DOI:** 10.1038/bjc.1991.217

**Published:** 1991-06

**Authors:** V. Laurence, M. A. Forbes, E. H. Cooper

**Affiliations:** Regional Radiotherapy Centre, Cookridge Hospital, Leeds, UK.

## Abstract

A study of the epithelial mucin marker MCA was made in 233 patients with breast cancer. Only 6% of 72 patients with Stage I-III disease had a raised MCA (greater than 15 U ml-1) when assessed following surgical treatment of the primary tumour. Raised levels of MCA occurred in one out of 20 (10%) patients with stable local recurrence, and six out of ten (60%) patients with progressive local recurrence. In 115 patients with metastases 89 (77%) had a raised MCA, tumour extent and disease activity both influenced the MCA level. The change of MCA level during the treatment of 11 cases of local recurrence and 55 cases of metastatic disease showed a 64 and 84% concordance respectively with the change in clinical status. Coincidental measurement of MCA and bone scans showed a raised MCA in one out of 63 (1.5%) patients with negative or equivocal scans, and 26 out of 35 (74%) with positive scans. MCA provides a useful marker for the monitoring of the treatment of local recurrence and metastatic disease, and an independent indicator of the effects of changes in treatment.


Br. J. Cancer (1991), 63, 1000–1004 © Macmillan Press Ltd., 1991

### Use of mucin like cancer associated antigen (MCA) in the management of breast cancer

V. Laurence[1], M.A. Forbes[2] & E.H. Cooper[2]

[1]*Regional Radiotherapy Centre, Cookridge Hospital, Leeds LS16 6QB;* [2]*Diagnostic Development Unit, Department of Chemical Pathology, University of Leeds, Leeds, UK.*

**Summary** A study of the epithelial mucin marker MCA was made in 233 patients with breast cancer. Only 6% of 72 patients with Stage I–III disease had a raised MCA (>15 U $ml^{-1}$) when assessed following surgical treatment of the primary tumour. Raised levels of MCA occurred in one out of 20 (10%) patients with stable local recurrence, and six out of ten (60%) patients with progressive local recurrence. In 115 patients with metastases 89 (77%) had a raised MCA, tumour extent and disease activity both influenced the MCA level. The change of MCA level during the treatment of 11 cases of local recurrence and 55 cases of metastatic disease showed a 64 and 84% concordance respectively with the change in clinical status. Coincidental measurement of MCA and bone scans showed a raised MCA in one out of 63 (1.5%) patients with negative or equivocal scans, and 26 out of 35 (74%) with positive scans. MCA provides a useful marker for the monitoring of the treatment of local recurrence and metastatic disease, and an independent indicator of the effects of changes in treatment.

Polymorphic epithelial mucins (PEM) are surface glycoproteins secreted by normal epithelia in large amounts into the external environment of the body. The loss of polarity and the disruption of the cellular barriers in cancer results in PEMs being shed into the internal environment and appear in increased concentration in the circulation (Hilgers *et al.*, 1988).

Several assays based on the detection of epitopes on PEMs have been described and some of them have been shown to be potentially useful as tumour markers in breast cancer. Human milk fat globule proteins and human breast cancer cells have been used to raise monoclonal antisera that can react with cancer associated PEMs both in tissues and in the plasma.

Several commerical assays have been developed to measure PEMs in breast cancer; the first was CA15.3 (Hayes *et al.*, 1986) and this has been followed by mucin like cancer associated antigen [MCA], (Stahli *et al.*, 1989), CA549 (Bray *et al.*, 1987), CA M26 and CA M29 (van Kamp *et al.*, 1989). So far the tests appear to have comparable sensitivities for the detection of systemic breast cancer and all are more sensitive than carcinoembryonic antigen. With the exception of CA15.3 there are few reports of how these markers perform in the routine clinical management of breast cancer.

The preliminary reports on MCA have indicated that its clinical value was most likely to be in the monitoring of recurrent and metastatic disease (Bieglmayer *et al.*, 1988; Cooper *et al.*, 1989; Rasoul-Rockenschaub *et al.*, 1989).

In this paper we report the results of a study investigating the relationship between serum MCA levels and the clinical status of over 200 patients attending a regional radiotherapy centre for the management and follow-up of post-operative breast cancer. We report on the use of serial measurements of MCA in patients receiving treatment for local and disseminated recurrent disease, and on the use of the marker as an adjunct to isotope bone scanning.

### Patients and methods

Serum samples were collected from 233 patients with breast cancer attending Cookridge Hospital, Leeds, between July 1988 and December 1989 (231 women and two men). There were three patient groups: 72 patients attending for post-operative radiotherapy after primary local excision or mastectomy; 128 patients with local or distant recurrence attending for assessment and treatment with hormones, cytotoxic chemotherapy and/or radiotherapy; and finally 33 patients on routine follow-up who had symptoms requiring investigation to exclude relapse.

Sera was collected from all patients at first assessment. Sequential samples were obtained from 66 patients receiving treatment who were under regular review. All serum samples were stored at −20°C until use. Patient information was not available at the time of serum marker assay, all samples being coded.

Data were analysed at the end of the study period using clinical information obtained from patients' notes, and blind, from marker values. Patient groups were subdividied further prior to analysis (see below).

### *Post-operative patients*

Patients were staged according to the standard UICC (International Union Against Cancer) breast cancer classification system. All patients had limited staging with chest X-ray, routine haematology and biochemistry screening. In addition, some had isotope bone scans, but this was not routinely performed. There were 28 patients with Stage 1, 32 with Stage 2, and 12 with Stage 3 disease. One patient was subsequently found to have asymptomatic bony metastases on a staging bone scan, one later developed local recurrence and one developed widespread metastatic disease on follow-up. These three patients have been used twice in the analysis (with initial marker value in the post-operative disease group and new marker value at the time of known change in status).

### *Local recurrence*

Twenty-nine patients had locally recurrent disease of the breast, chest wall or regional nodes. Four of these patients developed distant metastases later in the study and have been included in both groupings in the analysis (with marker values at entry and then at relapse). Thirty patients with local recurrence were thus available for analysis at the completion of the study. We subdivided the group retrospectively using information from their notes and without knowledge of marker levels. Twenty patients were described as having 'stable disease' (S.D). Their lesions were static or responding to treatment according to standard UICC criteria for a minimum of 3 months after study entry and they remained alive for a minimum of 6 months. Ten patients in this group

Received 6 August 1990; and in revised form 3 January 1991.

had 'progressive disease' (P.D.). Their lesions progressed despite treatment or they died within 6 months of entering the study.

*Metastatic disease*

One hundred and fifteen patients in the study had distant metastases. Ninety-nine had known metastatic disease at study entry of whom 39 were continuing with established treatment, 45 were commencing new treatment and 15 were having new symptoms investigated. Ten patients on routine follow-up found to have newly positive bone scans, together with the six previously mentioned patients in the post-operative and local recurrence groups, who subsequently developed metastases, are also included in this group.

We sub-divided these patients according to known site, tumour burden and disease activity at study entry. Any patient with metastases in one site only was defined as having 'single site' disease whereas those with metastases in more than one site were said to have 'multi-site' disease. Burden described the volume of known disease. 'Low burden' (L.B.) was defined as a single area within one site, such as one area of increased activity on an isotope bone scan, or a single pulmonary nodule on the chest X-ray. All other patients were defined as having 'high burden' (H.B.) disease. 'Activity' described the symptoms and response to treatment at study entry. Those who were asymptomatic and/or responding to treatment were said to have 'inactive disease' whereas those with symptoms and/or progressive disease were said to have 'active disease'. Assessment of clinical response was according to standard UICC criteria (Hayward *et al.*, 1977) by the patients' attending clinician without knowledge of marker values. External review of response was not obtained.

*Serial measurement of MCA levels in patients receiving treatment*

Sequential samples were obtained from a group of 55 patients with metastatic disease and 11 with local recurrence, all of whom were receiving treatment with hormones, chemotherapy and/or radiotherapy over 1 to 9 months (mean 3 months). Samples were taken monthly where practicable from patients on chemotherapy and at each attendance for follow-up from the rest (with a mean of three samples per patient; range two to seven). The responses to treatment as documented by the attending clinicians were compared to the patients' changing MCA values over the same time period. Disease status was defined as stable, progressive or responding according to standard UICC criteria. A change of marker level of ± 20% over 6 months (or equivalent rate of change) was considered to be significant.

*Bone scans*

Ninety-eight patients had isotope bone scans performed during the study period for staging or as part of symptom assessment. Serum samples were taken at the time of scanning and independently evaluated. All scans were reported by the patients own clinician to whom MCA values were unavailable.

Scans were classified as negative, positive or equivocal. Positive scans were defined as 'low burden' (L.B.) if only one site of increased activity was demonstrated and 'high burden' (H.B.) for all others. The 13 equivocal scans were supplemented by plain X-rays and serum alkaline phosphatase measurements. Eight were thought to represent degenerative change, one to represent low burden metastases and four to be of uncertain significance by the reporting clinicians.

*MCA assay*

The MCA-EIA (Roche Diagnostics, Welwyn Garden City, Herts) was used according to the manufacturer's instructions. The kit is a two-step solid phase enzyme immunoassay. The assay employs the same monoclonal mouse antibody to MCA (MAb b-12) in both positions of the sandwich (as capture antibody and as detection antibody), as this antibody recognises a repetitive binding site on the MCA molecule. The inter-assay CV was 9.6% and the intra-assay 8.5%.

*Statistical analysis*

The Mann Whitney test was used to compare differences between the groups.

### Results

A previous study of the MCA levels in 63 tumour-free breast cancer patients, 3–9 years after treatment of primary disease, showed a median and range of 7.4 U $ml^{-1}$ (1–21.1) and was similar to the levels found in healthy controls (Cooper *et al.*, 1989). Based on these results, the upper limit of normal MCA in disease-free women was set at 15 U $ml^{-1}$ (mean + 2 s.d.). The marker displays a non-Gaussian distribution and follows a log-normal pattern.

The results of initial marker values for the groups previously described above are shown in Table I. MCA levels of $>15$ U $ml^{-1}$ were present in 6% of patients with Stage I–III disease, 27% with local recurrence and 77% of those with metastatic disease. The ranges, median values and interquartile ranges for these groups and their subgroupings are shown in Figure 1. These were found to be significantly different ($P<0.05$) between the three main groupings.

**Table I** MCA levels in various patient groups

| *Patient group* | *Number of patients* | *Median MCA value U ml⁻¹* | *Range of MCA values U ml⁻¹* | *Number of patients with MCA > 15 U ml⁻¹* | *% with MCA > 15 U ml⁻¹* |
|---|---|---|---|---|---|
| *Post-operative* | | | | | |
| Stage I | 28 | 7 | 0.7–16.5 | 1 | 4 |
| Stage II | 32 | 8.6 | 0.7–18 | 2 | 6 |
| Stage III | 12 | 11.6 | 1.1–21.5 | 1 | 8 |
| All | 72 | 8 | 0.7–21.5 | 4 | 6 |
| *Local recurrence* | | | | | |
| Stable disease | 20 | 9.3 | 2–77 | 2 | 10 |
| Progressive disease | 10 | 21.5 | 10.5–77 | 6 | 60 |
| All | 30 | 11 | 2–77 | 8 | 27 |
| *Metastatic disease* | | | | | |
| Low burden inactive | 8 | 7.5 | 1.5–17.5 | 1 | 12.5 |
| Low burden active | 17 | 15.8 | 2–47 | 9 | 53 |
| High burden inactive | 17 | 22 | 3.8–22 | 12 | 72 |
| High burden active | 73 | 75 | 6.3–2500 | 67 | 92 |
| All | 115 | 38 | 1.5–2500 | 89 | 77 |

**Figure 1** Distribution of MCA levels in patients following surgical treatment of primary breast cancer, with local recurrence and with distant metastases. LBi = Low burden inactive; HBi = High burden inactive; LBa = Low burden active; HBa = High burden active; SD = Stable disease; PD = Progressive disease; Horizontal line = 15 U ml$^{-1}$ [the upper limit of the normal range].

In the post-operative group of patients with Stage I, II and III disease, 4%, 6% and 8% respectively had raised marker values. This represents a significant upwards trend for increasing MCA value with increasing disease stage. Of the four patients with raised markers two remained tumour free at 9 and 11 months follow-up (MCA values of 16 and 18 U ml$^{-1}$); one remained tumour free with falling markers at 3 months follow-up (from 16.5 to 13.5 U ml$^{-1}$), and the fourth patient with a value at the top of the range of 21.5 U ml$^{-1}$ was subsequently found to have asymptomatic bony metastases. There were three further patients with MCA values at the top of the normal range of 15 U ml$^{-1}$. Of these one remains well, one has developed local recurrence (the only one from the post-operative group) with rising titres of MCA and the third complained of flitting bone pains 2 months after operation and on isotope bone scanning was shown to have bony metastases. She died of widespread disease 3 months later. Her MCA value was 18 U ml$^{-1}$ 1 week after the first sample and 103 U ml$^{-1}$ at the time of her bone scan.

Of the 30 patients with local recurrence, 27% had raised MCA values with a median of 11 U ml$^{-1}$ and a range of 2–77 U ml$^{-1}$. Within this group those with progressive disease had significantly higher values than those with stable disease ($P = 0.001$). Seventy-seven per cent of the 115 patients with metastatic disease had raised markers with a median value of 38 U ml$^{-1}$ and range of 1.5–2,500 U ml$^{-1}$. As shown in Figure 1, the greater the burden and activity the higher the markers tend to be ($P = 0.001$ for high burden against low burden).

Table II shows the numbers of patients with metastatic disease as described by single or multiple site (i.e. more than one site) and their MCA values. Most patients in the study had either bony or multi-site metastases. Those with multi-site disease had significantly higher marker values than those with single sites ($P = 0.001$). There was no significant difference between marker values according to individual site.

*Correlation of changing MCA levels with the clinical course of disease.*

Sequential samples were obtained from 66 patients receiving treatment. Figure 2 shows the changing values over 2–8 months for the 55 patients with metastatic disease. Table III shows the concordance values. Eighty-four percent of those patients with metastatic disease and 64% of the 11 patients with local recurrence had changing markers which corresponded to their clinical status. Those with responding or progressing metastatic disease had the highest concordance. Those with clinically stable disease, but rising or falling markers may be showing a lead time effect which will become apparent on further long-term follow-up.

Figures 3 to 6 show the graphs of four individual patients as examples of the MCA changes occurring over time. Patient 1 had known metastatic disease in a number of sites and was receiving single agent weekly chemotherapy at the start of the study. In February 1989 her symptoms progressed and her treatment was changed. Despite this, her condition deteriorated and she died in April 1989. As can be seen from the graph, her MCA values over this time period show a progressive rise. In comparision, Patient 2, a man with nodal, pleural, and bony metastases, previously treated with hormones, commenced weekly single agent chemotherapy in January 1989 with a good symptomatic and objective partial response which continued until July 1989. This

**Figure 2** Rates of change of MCA in patients with metastatic disease on treatment according to response.

**Table II** MCA levels of patients with metastatic disease according to sites involved

| Site | Number of patients | Median MCA U ml$^{-1}$ | Range of MCA values U ml$^{-1}$ | Number of patients with MCA > 15 U ml$^{-1}$ | % with MCA > 15 U ml$^{-1}$ |
|---|---|---|---|---|---|
| Central nervous system | 3 | 17 | 5.6–27 | 2 | 67 |
| Liver | 4 | 23 | 6.3–169 | 3 | 75 |
| Lung/pleura | 12 | 27 | 1.5–1360 | 9 | 75 |
| Bone | 52 | 27 | 2–399 | 37 | 71 |
| Multi-site | 44 | 70 | 6–2500 | 40 | 91 |

**Table III** Concordance of change of MCA level and clinical status

| | *Clinical status* | *Number of patients* | *Appropriate MCA changes number* | *Concordance %* |
|---|---|---|---|---|
| | Progression | 26 | 24 | 92 |
| Metastatic | Stable | 15 | 10 | 67 |
| disease | Responding | 14 | 12 | 86 |
| | All | 55 | 46 | 84 |
| | Progression | 3 | 1 | 33 |
| Local | Stable | 5 | 3 | 60 |
| recurrence | Responding | 3 | 3 | 100 |
| | All | 11 | 7 | 64 |
| Total | | 66 | 53 | 80 |

**Figure 3** Progressive rise of MCA level in a patient with widespread metastatic disease.

**Figure 4** Symptomatic improvement in a male patient after changing from hormone to chemotherapy with corresponding fall of MCA.

**Figure 5** Fall of MCA following a change of chemotherapy in metastatic disease.

**Figure 6** Change of MCA level in a patient with a local recurrence following post-operative radiotherapy for Stage II disease that was not controlled by chemotherapy.

was mirrored by the falling MCA titres. Patient 3 had progressive disease which responded to a change in treatment before progressing. Patient 4 was initially treated with radiotherapy and adjuvant chemotherapy for Stage II disease. She developed local recurrence within 6 months and has progressive local disease despite further chemotherapy.

### *Bone scans*

As described above, 98 patients had marker levels assayed at the time of assessment or staging with isotope bone scan. The results are shown in Table IV. Only one patient (1.5%) with a negative or equivocal scan had a raised MCA level (16.5 U $ml^{-1}$) compared to 26 out of 35 patients (74%) with positive scans ($P = 0.001$). The predictive value of a raised MCA for a positive diagnosis was 96% and of a normal MCA for a negative diagnosis 87%.

### Discussion

A study of the MCA levels in women who have remained tumour-free for several years after treatment of primary breast cancer showed that the median and range is similar to that of normal controls. Repeated annual measurements in these patients show a narrow intra-patient variation ($< \pm 2.0$ U $ml^{-1}$) even though the overall range was wide (1–21 U $ml^{-1}$) and therefore interpretation of MCA changes is easier if the patients' own tumour-free level is known (Cooper *et al.*, 1989).

For convenience we have used 15 U $ml^{-1}$ as the upper-limit of normal, which is close to 14 U $ml^{-1}$ and 14.4 U $ml^{-1}$ adopted by Bieglmayer *et al.* (1988), and Rasoul-Rochenschaub *et al.* (1989), but is higher than the 11 U $ml^{-1}$ advised by the manufacturers. In earlier reports of MCA levels in tumour free patients, Rasoul-Rochenschaub *et al.* (1989), reported in 263 patients the mean ± s.e.m. to be $4.5 \pm 0.3$ U $ml^{-1}$ and Cooper *et al.* (1989) found a median of 7.4 U $ml^{-1}$ in 63 patients. Hence it is difficult to draw conclusions about the significance of the post-operative levels when they are based on a single reading. Three patients in the current series showed evidence of recurrent or metastatic disease within 6 months of their post-operative assessment, their MCA levels at the time of initial assessment being 15, 15 and 21.5 U $ml^{-1}$ respectively. Bieglmayer *et al.* (1988), studied the change in MCA levels in 49 high risk patients, 20% of whom developed metastases during the observation period. A rising MCA level was found to precede metastases in 91% with a lead time of 1–15 months.

As the information on the use of breast cancer markers in clinical practice increases it becomes clear that the epithelial mucin markers CA15.3 and MCA have similar characteristics in terms of their ability to reflect the progress of metastatic

Table IV MCA levels at the time of isotope bone scanning

| *Scan result* | *Number of patients* | *Median MCA U $ml^{-1}$* | *Range of MCA U $ml^{-1}$* | *Number with MCA > 15 U $ml^{-1}$* | *% with MCA > 15 U $ml^{-1}$* |
|---|---|---|---|---|---|
| Scan negative | 50 | 7 | 0.6–16.5 | 1 | 2 |
| Scan equivocal | 13 | 7.7 | 2–13.6 | 0 | 0 |
| *Scan positive* | | | | | |
| Low burden | 8 | 13.7 | 2.4–36.6 | 4 | 50 |
| High burden | 27 | 42 | 3.8–333 | 22 | 81 |

cancer (Bieglmayer *et al.*, 1988; Rasoul-Rockenschaub *et al.*, 1989; Eskelinen *et al.*, 1989; Colomer *et al.*, 1989). When a cut-off level is used based on controls or tumour free patients it is evident that the percentage of raised markers in metastatic disease studied vertically is influenced by the site, and disease activity as shown in Tables I and II. In general normal levels are more likely to occur when there is a low tumour burden or inactive disease. A similar conclusion was drawn by Colmer *et al.* (1989) in their study of CA15.3. In a comparison of skeletal lesions with progressive and stable disease the mean ± s.e.m. MCA levels were 39.3 ± 1.9 and 21 ± 1.0 U $ml^{-1}$ and for visceral lesions the levels were 49.3 ± 2.8 and 19.8 ± 1.0 U $ml^{-1}$ respectively (Rasoul-Rockenschaub *et al.*, 1989). Comparable trends are shown in our present study and that reported by Bieglmayer *et al.* (1989) and Steger *et al.* (1989).

It appears that none of the mucin breast cancer markers are sensitive to small tumour volumes in soft tissues, as shown by primary tumours or localised recurrence (Cooper *et al.*, 1989; Bieglmayer *et al.*, 1989; Bon *et al.*, 1990). However, in local recurrence the more extensive and aggressive lesions tended to have a raised MCA in our series.

It is clear from the relatively short term follow-up studies shown in this report that it is the change of MCA level that provides the most useful data for routine patient management. There is a high concordance between the rate of change of the marker levels and clinical and imaging evidence of tumour progression, regression or stability. If measurements are made during the first 2–3 weeks after a change of treatment, the levels of the mucin markers may show the expected rise during progression or fall during regression, but an initial parodoxical surge can occur in the good responders (Kiang *et al.*, 1990). The measurement of MCA at the time of performing a bone scan provides an adjunct to the interpretation of the scan. In this series, raised MCA levels were only seen in 1.5% of patients with negative and equivocal scans while 74% of those with positive scans had raised values.

Although MCA lacks the specificity and sensitivity to make it an ideal cancer marker for breast cancer, it performs better than CEA (Rasoul-Rockenschaub *et al.*, 1989; Beiglmayer *et al.*, 1989; Steger *et al.*, 1989) as do the other epithelial mucin breast cancer markers (Bon *et al.*, 1990). Our experience of using the test in a regional radiotherapy centre suggests that MCA measurements can be helpful to the clinician in the following circumstances. At presentation it is valuable to obtain an initial value as an adjunct to staging and to provide a reference value for subsequent monitoring; at this time high values can draw attention to asymptomatic metastatic disease and warrant study by bone and liver scans. In metastatic disease the change of level of MCA is helpful in assessing progress in patients without measurable lesions.

When there is doubt about the interpretation of a bone scan such as a pattern of activity untypical of metastases, then the marker level may help to distinguish degenerative from metastatic disease. Similarly, a change in MCA level may reinforce the clinical opinion that symptoms during the follow-up period are likely to be due to recurrent disease prompting further investigation. It must be kept in mind that the mucin breast cancer markers can be raised in other tumours such as those of the ovary, GI tract and lung (Kenemans *et al.*, 1988), as well as benign liver, and autoimmune disease such as cirrhosis and SLE. An unexpected rise in nadir must not be assumed to be due to recurrent breast cancer but needs full evaluation. The markers, no doubt, can be used as one parameter in the assessment of the effects of new treatment regimes.

We would like to thank Drs Ash, Cartwright, Close, Jones, Rothwell and Taylor for allowing their patients to be part of this study. We are also grateful to the Nursing Staff at Cookridge Hospital and the Nuclear Medicine Department for their assistance in obtaining samples.

### References

BIEGLMAYER, C., SZEPESI, T. & NEUNTEUFEL, W. (1988). Follow-up of metastatic breast cancer with a mucin-like carcinoma-associated antigen: comparison to CA15.3 and carcinoembryonic antigen. *Cancer Lett*, **42**, 199.

BIEGLMAYER, C., SZEPESI, T., NEUNTEUFEL, W. & SCHIEDER, K. (1989). Properties of MCA and surveillance of breast cancer patients with tumor markers. In *Human Tumor Markers*. Ting, S.W., Chen, J.S. & Schwartz, M.K. (eds). Elsevier Science Publishers: Amsterdam.

BON, G.G., KENEMANS, P., VAN KAMP, G.J., YEDEMA, C.A. & HILGERS, J. (1990). Review on the clinical value of polymorphic epithelial mucin tumor markers for the management of carcinoma patients. *J. Nucl. Med. Allied Sci.*, **34**, 151.

BRAY, K.R., KODA, J.E. & GAUR, P.K. (1987). Serum levels and biochemistry characteristics of a cancer associated antigen CA 549, a circulating breast cancer marker. *Cancer Res.*, **47**, 5853.

COLOMER, R., RUIBAL, A. & SALVADOR, L. (1989). Circulating tumour marker levels in advanced breast carcinoma correlate with extent of metastatic disease. *Cancer*, **64**, 1774.

COOPER, E.H., FORBES, M.A., HANCOCK, A.K., PRICE, J.J. & PARKER, D. (1989). An evaluation of mucin-like carcinoma associated antigen (MCA) in breast cancer. *Br. J. Cancer*, **59**, 797.

ESKELINEN, M., TIKANOJA, S. & COLLAN, Y. (1989). Efficient test for cancer antigens: decreased levels of cancer antigen in serum after excision of breast tumor. *Anticancer Res.*, **9**, 437.

HAYES, D.F., ZURAWSKI, V.R. & KUFE, D.W. (1986). Comparison of circulating CA15.3 and CEA levels in patients with breast cancer. *J. Clin. Oncol.*, **4**, 1542.

HAYWARD, J.L., CARBONE, P.P., HEUSON, J.C., KUMAOKA, S., SEGALOFF, A. & RUBENS, R.D. (1977). Assessment of response to therapy in advanced breast cancer: a project of the Programme on Clinical Oncology of the International Union Against Cancer. *Cancer*, **39**, 1289.

HILGERS, J., ZOTTER, S. & KENEMANS, P. (1988). Polymorphic epithelial mucin and CA125-bearing glycoprotein in basic and applied carcinoma research. *Cancer Res.*, **11–12**, 3.

KENEMANS, P., BAST, R.C. Jr., YEDEMA, C.A., PRICE, M.R. & HILGERS, J. (1988). CA125 and polymorphic epithelial mucin as serum tumor markers. *Cancer Rev.*, **11–12**, 119.

KIANG, D.T., GREENBERG, L.J. & KENNEDY, B.J. (1990). Tumor marker kinetics in the monitoring of breast cancer. *Cancer*, **65**, 193.

RASOUL-ROCKENSCHAUB, S., ZIELINSKI, C.C., KUBISTA, E. & 6 others (1989). Diagnostic values of mucin-like antigen (MCA) in breast cancer. *Eur. J. Cancer Clin. Oncol.*, **25**, 1067.

STAHLI, C., CARAVATTI, M., TAKACKS, B., ANDRES, R. & CARMAN, H. (1989). A mucinous carcinoma associated antigen MCA defined by three MAb against different epitopes. *Can. Res.*, **48**, 6799.

STEGER, G.G., MADER, R., DERFLER, K., MOSER, K. & DITTRICH, C. (1989). Mucin-like cancer associated antigen (MCA) compared with CA15-3 in advanced breast cancer. *Klin Wochenschr.*, **67**, 813.

VAN KAMP, G.J., YEDEMA, K.KA., KOK, A., POORT, R., HILGERS, J. & KENEMANS, P. (1989). Evaluation of an EIA Kit for carcinoma-associated mucin antigens CA M26 and CA M29. *J. Tumour Marker Oncol.*, **4**, 363.


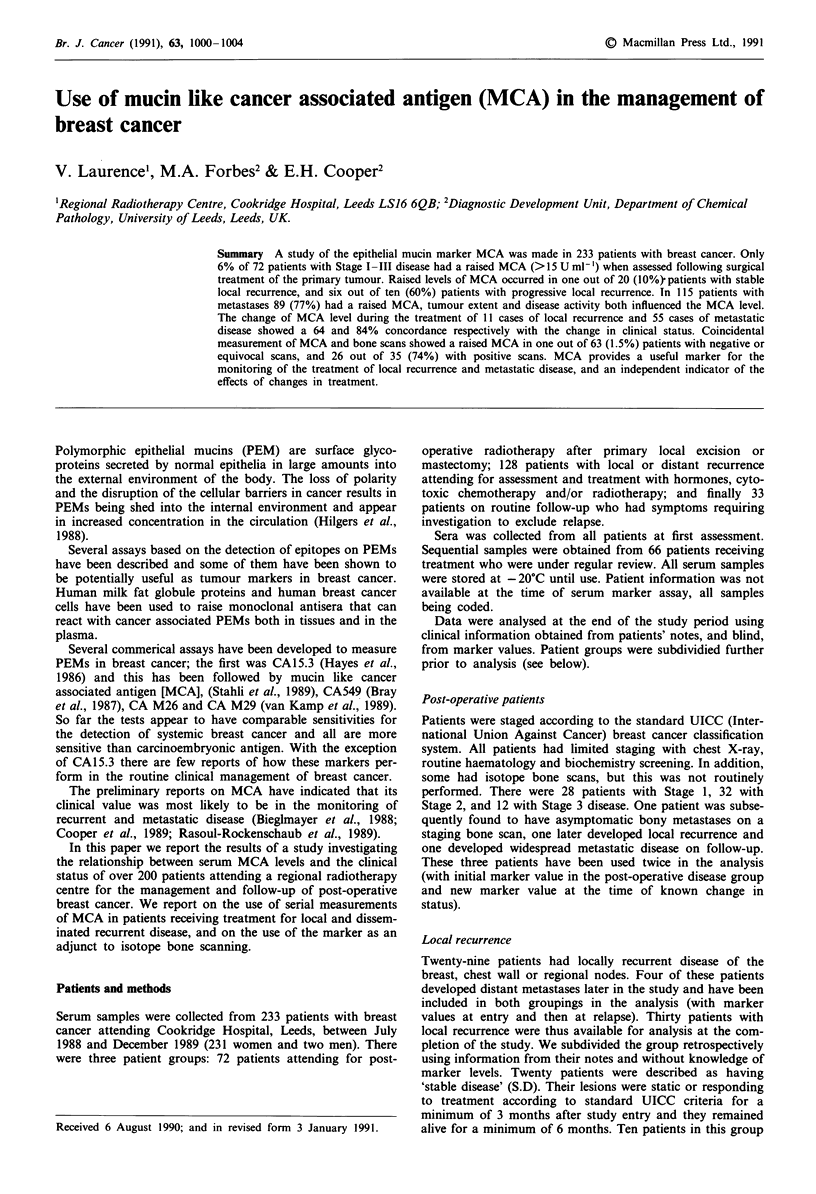

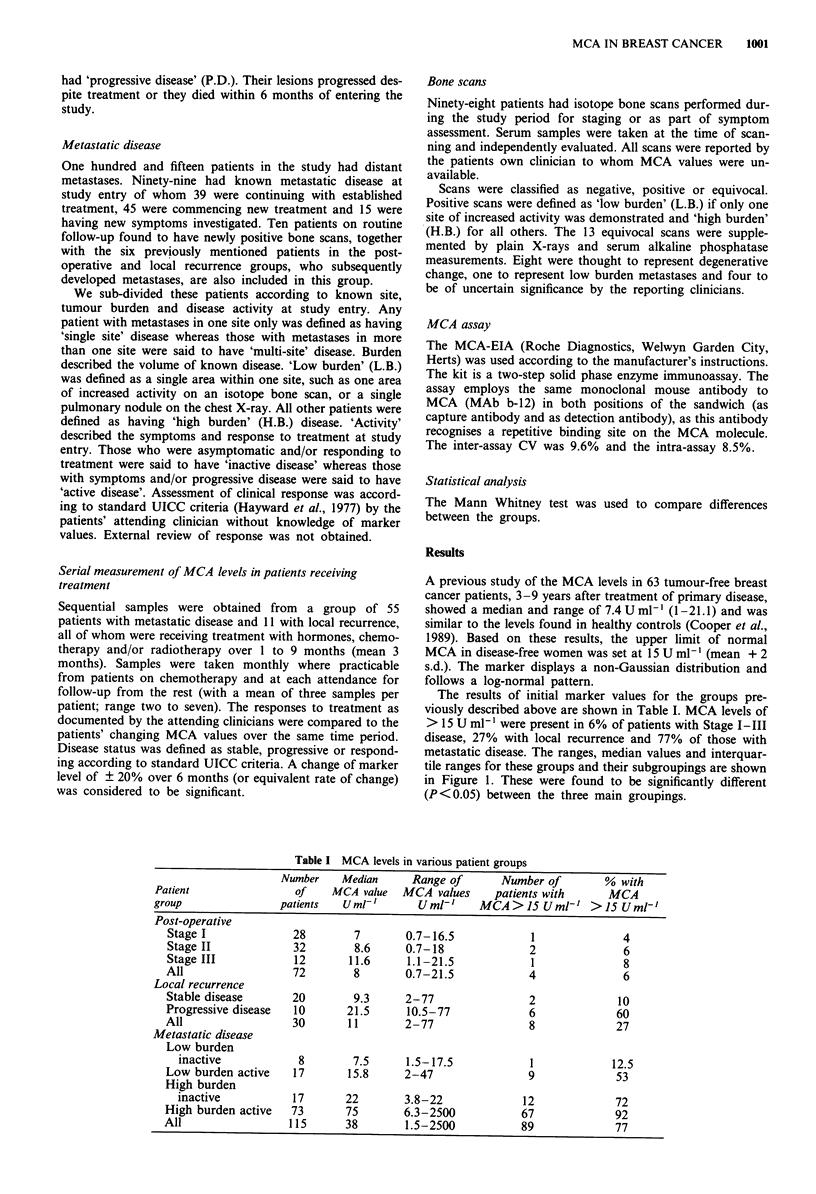

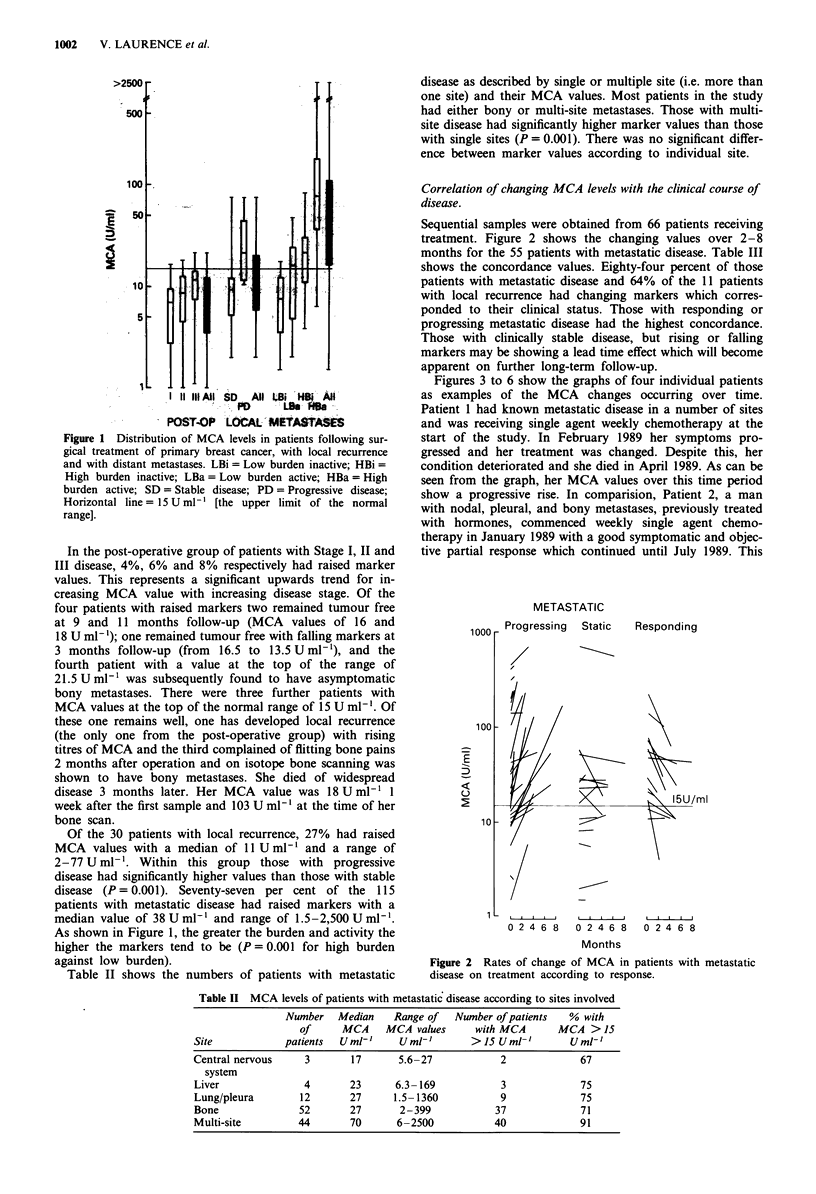

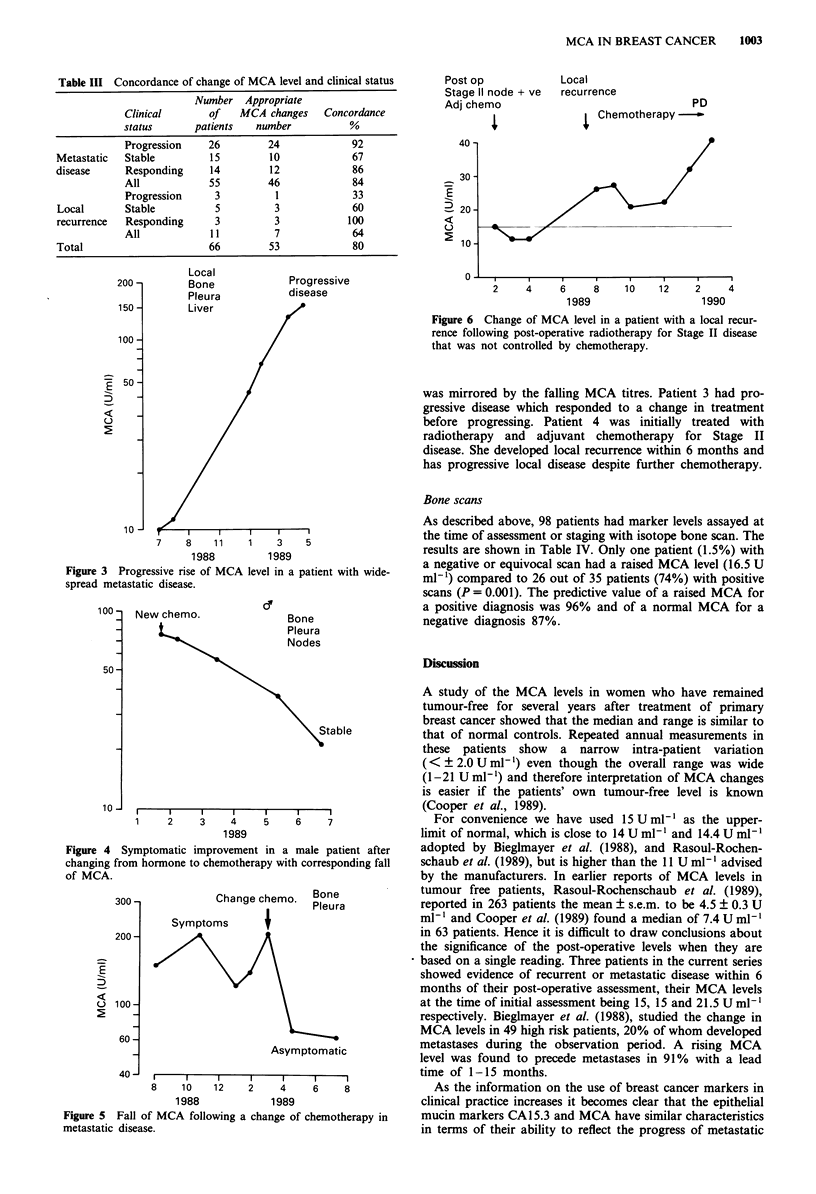

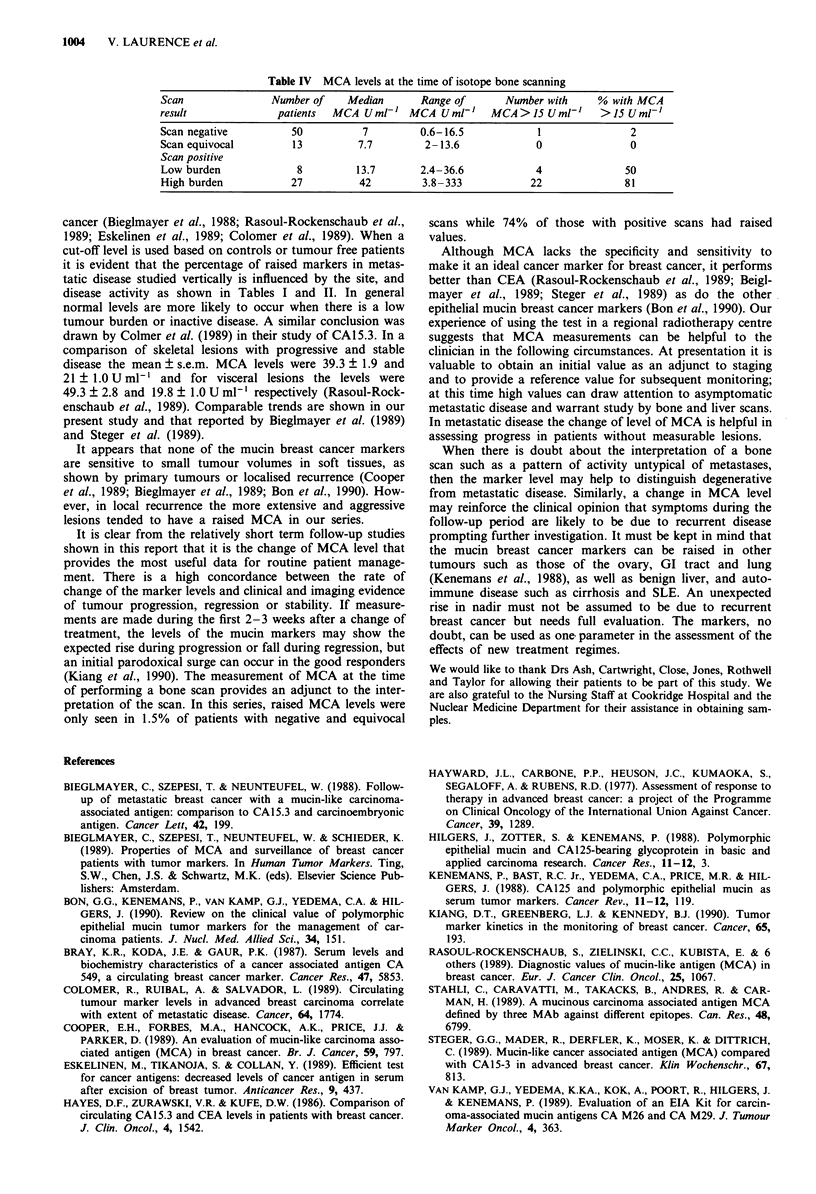


## References

[OCR_00715] Bieglmayer C., Szepesi T., Neunteufel W. (1988). Follow-up of metastatic breast cancer patients with a mucin-like carcinoma-associated antigen: comparison to CA 15.3 and carcinoembryonic antigen.. Cancer Lett.

[OCR_00734] Bray K. R., Koda J. E., Gaur P. K. (1987). Serum levels and biochemical characteristics of cancer-associated antigen CA-549, a circulating breast cancer marker.. Cancer Res.

[OCR_00743] Cooper E. H., Forbes M. A., Hancock A. K., Price J. J., Parker D. (1989). An evaluation of mucin-like carcinoma associated antigen (MCA) in breast cancer.. Br J Cancer.

[OCR_00747] Eskelinen M., Tikanoja S., Collan Y. (1989). Efficient test for cancer antigens: decreased levels of cancer antigen in serum after excision of breast tumor.. Anticancer Res.

[OCR_00752] Hayes D. F., Zurawski V. R., Kufe D. W. (1986). Comparison of circulating CA15-3 and carcinoembryonic antigen levels in patients with breast cancer.. J Clin Oncol.

[OCR_00757] Hayward J. L., Carbone P. P., Heuson J. C., Kumaoka S., Segaloff A., Rubens R. D. (1977). Assessment of response to therapy in advanced breast cancer: a project of the Programme on Clinical Oncology of the International Union Against Cancer, Geneva, Switzerland.. Cancer.

[OCR_00774] Kiang D. T., Greenberg L. J., Kennedy B. J. (1990). Tumor marker kinetics in the monitoring of breast cancer.. Cancer.

[OCR_00730] Marino P., Buccheri G., Preatoni A., Ferrigno D., Cori P., Rosti A., Mozzi R., Moroni G. A. (1990). Soluble interleukin-2 receptor: a new prognostic marker in lung cancer.. J Nucl Med Allied Sci.

[OCR_00779] Rasoul-Rockenschaub S., Zielinski C. C., Kubista E., Vavra N., Pospischil E., Staffen A., Czerwenka K., Aiginger P., Spona J. (1989). Diagnostic value of mucin-like carcinoma-associated antigen (MCA) in breast cancer.. Eur J Cancer Clin Oncol.

[OCR_00790] Steger G. G., Mader R., Derfler K., Moser K., Dittrich C. (1989). Mucin-like cancer-associated antigen (MCA) compared with CA 15-3 in advanced breast cancer.. Klin Wochenschr.

